# Venous Sinus Stenting for Challenging Cases of Idiopathic Intracranial Hypertension: A Case Series From a Tertiary Care Center in Riyadh, Saudi Arabia

**DOI:** 10.7759/cureus.83534

**Published:** 2025-05-05

**Authors:** Bashaier G AlQahtani, Turki Bin Saqyan, Maher Sahnoun

**Affiliations:** 1 Neurology, Prince Sultan Military Medical City, Riyadh, SAU; 2 Neuroradiology, Prince Sultan Military Medical City, Riyadh, SAU

**Keywords:** case report series, idiopathic intracranial hypertension (iih), intracranial pressure (icp), kingdom of saudi arabia (ksa), venous sinus stenting (vss)

## Abstract

Idiopathic intracranial hypertension (IIH) is characterized by elevated intracranial pressure that causes headaches and visual disturbances. While medical management is the primary treatment, some patients remain refractory. Venous sinus stenting (VSS) has emerged as a potential alternative. This case series discusses the outcomes of VSS in patients with refractory IIH.

Patients with IIH who underwent VSS at a tertiary hospital in Riyadh were reviewed. The data collected included demographics, clinical presentation, diagnostic imaging findings, intraprocedural pressure measurements, and clinical and radiological outcomes before and after stenting. Verbal consent was obtained from all patients for participation in this case series.

All patients demonstrated transverse sinus stenosis and had persistent symptoms despite medical therapy. Following VSS, all patients experienced significant improvements in intracranial pressure gradients, papilledema grades, visual symptoms, and the frequency and intensity of headaches. No procedural complications occurred, although one patient experienced transient worsening of symptoms after the procedure.

This case series suggests that VSS may be an effective treatment for carefully selected patients with refractory IIH and can lead to improvements in clinical and radiological outcomes. However, larger, prospective, controlled studies with longer follow-ups are needed to confirm these findings and establish the long-term efficacy and safety of VSS.

## Introduction

Idiopathic intracranial hypertension (IIH) is a neurological disorder characterized by elevated intracranial pressure (ICP) without an identifiable cause. This condition predominantly affects women of childbearing age and is strongly associated with obesity [1,2]. The incidence of IIH ranges from 1 to 2 per 100,000 in the general population, but it may reach 20 per 100,000 among women with obesity [3]. If left untreated, IIH can lead to debilitating headaches, visual disturbances, and permanent vision loss [1,2]. Initial management typically involves weight loss, dietary modifications, and medical therapy, including carbonic anhydrase inhibitors (such as acetazolamide) and diuretics [4]. However, a significant proportion of patients experience persistent symptoms or intolerable side effects despite maximal medical management [5]. In refractory cases, invasive interventions such as cerebrospinal fluid (CSF) diversion procedures have been considered [6].

Venous sinus stenting (VSS) has emerged as a less invasive alternative for carefully selected patients with IIH who exhibit venous sinus stenosis [7]. This endovascular procedure involves placing a stent within the affected dural venous sinus, typically the transverse sinus, to mechanically widen the narrowed segment and improve cerebral venous outflow [8]. The underlying mechanism involves alleviating stenosis-induced venous outflow obstruction, thereby disrupting the ICP-venous hypertension feedback loop [9]. Several studies have suggested that VSS can effectively reduce intracranial pressure, improve visual outcomes, and alleviate headaches in appropriately selected patients [10-12]. Although some meta-analyses have indicated that VSS is associated with significant improvements in headache, papilledema, and visual outcomes [13], the long-term efficacy and safety of VSS remain areas of ongoing investigation [14].

This case series contributes to the existing body of evidence regarding VSS in the management of refractory IIH. We present the outcomes of four patients who underwent VSS at a tertiary care center in Riyadh, Saudi Arabia, with a focus on the potential benefits and limitations of the procedure in treating this challenging condition.

## Case presentation

The inclusion of cases in this study was consecutive, involving all eligible patients presenting between January 2023 and December 2024.

Case 1

A 20-year-old obese woman with a body mass index of 39 kg/m^2^ presented with pulsating right-sided headaches, photophobia, and blurred vision with pulsatile tinnitus lasting for four months prior to presentation. The initial MRI showed a prominent optic sheath complex bilaterally four months prior to presentation. The condition of the patient worsened, necessitating treatment in the emergency department. Examination revealed bilateral disc edema (grade IV papilledema (Frisén scale)) and binocular diplopia. Lumbar puncture revealed an opening pressure of 55 cm H_2_O. Diagnostic angiography revealed moderate dominant stenosis of the right transverse sinus.

**Figure 1:**
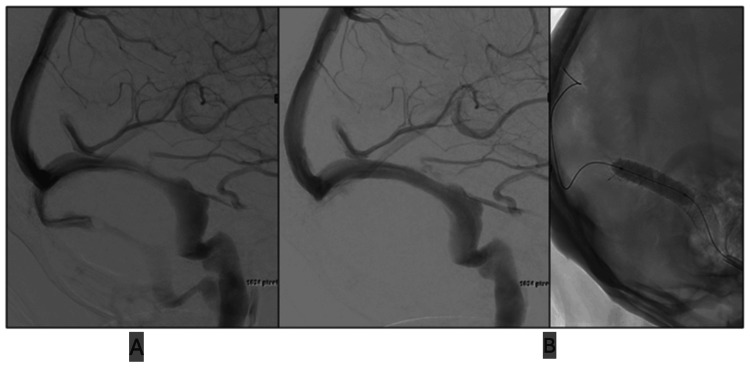
Lateral view venogram before (A) and after (B) stenting. Angioplasty with a 7 mm × 30 mm Sterling balloon into an 8 mm × 40 mm Casper stent

**Figure 2:**
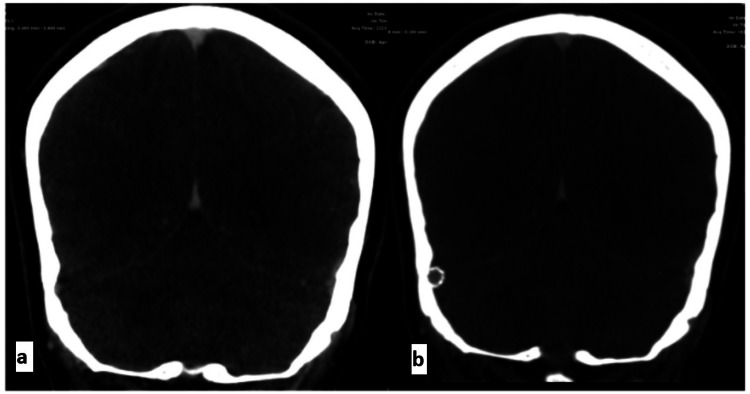
CTV before (a) and after (b) stenting showing good patency of the stent in the right transverse sinus CTV: computed tomographic venography

VSS was performed six months later using a Casper stent, an 8 × 40 mm stent (MicroVention, Inc., USA) with a Sterling balloon (7 x 30 mm) angioplasty (Boston Scientific, USA) (Figure 1). The pressure gradient improved from 36 cm H_2_O to 18 cm H_2_O after stenting. After the procedure, the patient was maintained on dual antiplatelet therapy (DAPT) and acetazolamide (1.5 g) twice daily, initiated before the procedure, and furosemide (40 mg) daily. Four weeks after stenting, her visual acuity improved to 20/20 in both eyes with normal visual fields in both eyes. Two months later, computed tomographic venography (CTV) demonstrated satisfactory stent patency (Figure 2).

Case 2

**Figure 3:**
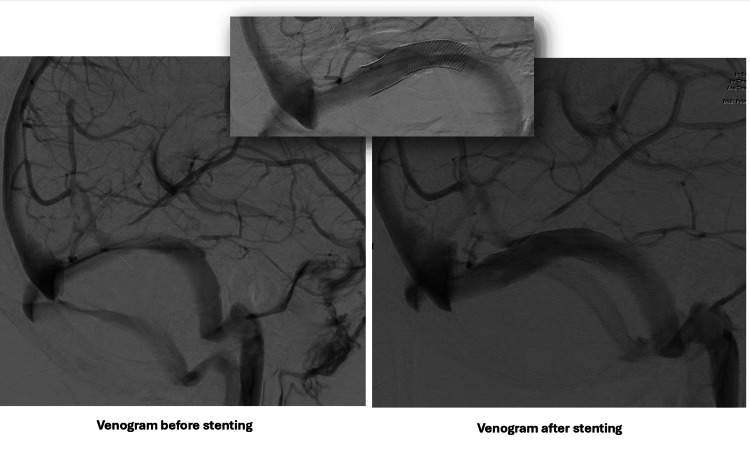
Cerebral angiogram

A 20-year-old obese man with a body mass index of 45 kg/m^2^ presented with vision changes and severe headaches lasting for one week before presentation. Examination revealed bilateral papilledema (grade IV), limited left eye abduction, and bitemporal visual field loss. CTV showed bilateral narrowing of the transverse venous sinuses. Diagnostic angiography confirmed dominant right transverse sinus stenosis (Figure 3).

Stenting was performed, which improved the pressure gradient from 38 to 20 cmH_2_O. After the procedure, the patient experienced temporary worsening of headaches and visual symptoms. The transient worsening of headaches and visual symptoms post-VSS lasted approximately for two days, likely due to hemodynamic changes.

Management involved close monitoring, symptomatic treatment with analgesics, and computed tomography (CT), which demonstrated stable optic nerve sheaths without new findings. Five months after stenting, there was no active papilledema, and the patient was off diuretics with no headache or visual symptoms.

Case 3

A 45-year-old woman with IIH presented with worsening symptoms for one month despite medical management. The patient's initial complaints included band-like headaches, painful eye movements, intermittent visual blurring, and perioral numbness. She was initially administered 750 mg of acetazolamide twice daily. The initial ophthalmological examination revealed a visual acuity of 6/6 oculus uterque (OU) and normal visual fields bilaterally, and fundoscopy showed slight supratemporal elevation of the right optic disc without frank papilledema. MR venography revealed bilateral transverse sinus stenosis without acute venous thrombosis.

**Figure 4:**
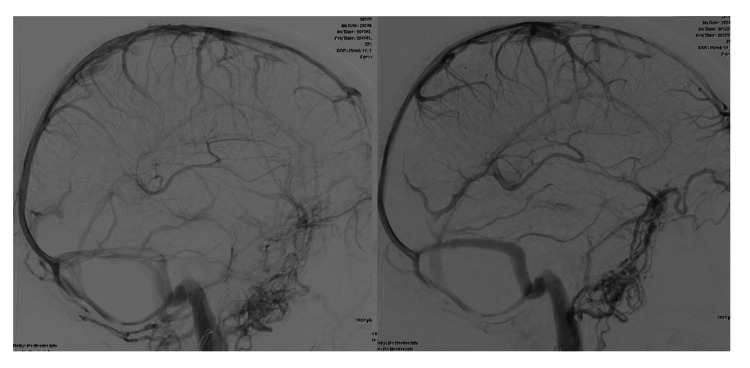
Cerebral angiograms in the venous phase with a lateral view comparing the flow in the right transverse sinus before (left) and after stenting (right). Notably, the occipital collaterals have disappeared

One month after the neurological consultation, the patient reported worsening symptoms, including exacerbation of headache for one week, periocular pressure sensation, visual obscurations, bilateral aural fullness, and headaches worsened by postural changes, which were partially alleviated using simple analgesics. Follow-up fundoscopy revealed grade-1 bilateral disc edema. Given the refractory nature of her symptoms, the patient underwent right transverse sinus stenting with angioplasty (Figure 4). The intraprocedural pressure measurements were a pre-stenting gradient of 27 cmH_2_O, a post-stenting gradient of 15 cmH_2_O, superior sagittal sinus pressures of 30 cmH_2_O (pre-stent) and 20 cmH_2_O (post-stent), and sigmoid sinus pressures of 3 cmH_2_O (pre-stent) and 5 cmH_2_O (post-stent).

Postprocedural management included prophylactic low-molecular-weight heparin for 10 days and continuation of DAPT for three months. During the two-week ophthalmological follow-up period, significant improvements were noted. The patient had a visual acuity of 20/20 OU and intraocular pressure of 14/13 mmHg, and fundoscopy indicated clear discs and maculae bilaterally with resolution of papilledema.

Case 4

**Figure 5:**
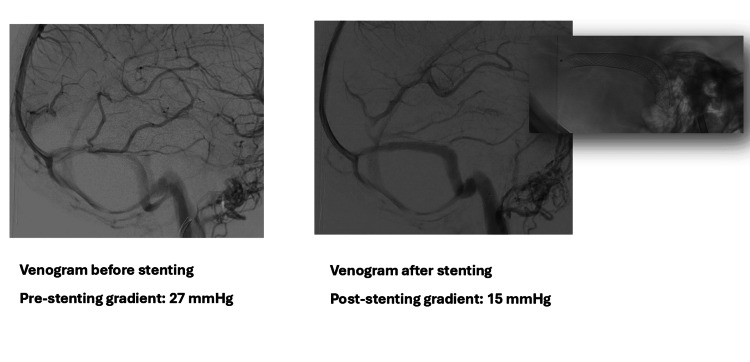
Cerebral angiograms in the venous phase with a lateral view comparing the flow in the right transverse sinus before and after stenting

**Figure 6:**
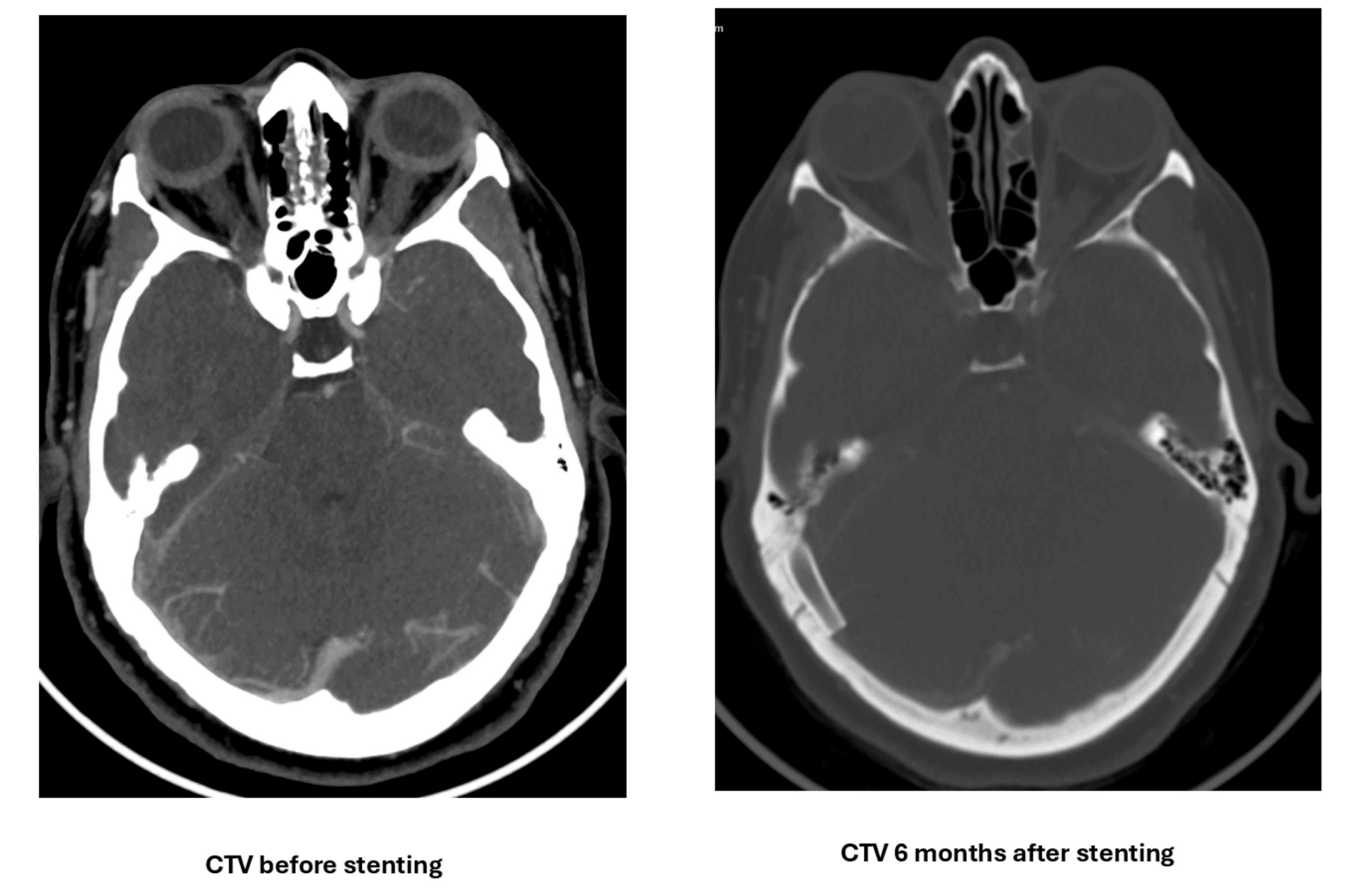
CTV before and after stenting showing good patency of the stent in the right transverse sinus CTV: computed tomographic venography

A 21-year-old woman with IIH after ventriculoperitoneal shunt removal presented with worsening headaches and visual symptoms lasting for two weeks prior to presentation. She was administered 500 mg acetazolamide three times daily and 40 mg furosemide once daily. Examination revealed bilateral papilledema (grade III, right; grade II, left). The CTV showed moderate-to-severe stenosis of the right transverse sinus. A cerebral angiogram confirmed dominant stenosis in the right transverse sinus (Figures 5-6).

Angioplasty with stenting of the right transverse sinus was performed. The pressure gradient decreased from 15 to 1.4 cmH_2_O after stenting. After the procedure, the patient experienced an improvement in papilledema and symptoms. The patient was treated with DAPT and acetazolamide. Currently, the patient is only taking aspirin.

## Discussion

This case series provides valuable insights into the efficacy of VSS in patients with IIH. The results demonstrated significant improvements in symptoms and clinical parameters of all patients who underwent VSS, particularly regarding intracranial pressure, papilledema, visual symptoms, and headaches. These findings align with those of several recent studies showing that VSS is an effective treatment option for IIH.

A systematic review and meta-analysis by Nicholson et al. corroborated our observations and reported high rates of improvement in papilledema (93.7%), headaches (79.6%), and pulsatile tinnitus (90.3%) after VSS [15]. Similarly, our case series showed marked improvement in papilledema grades and resolution of visual field defects post-stenting, consistent with the findings reported by Garner et al., Azzam et al., and Liu et al. in their respective studies [10-12].

The limitations of medical therapy in some patients with IIH were highlighted in the current study, because patients experienced persistent or worsening symptoms despite aggressive medical management. This finding highlights the necessity for alternative therapeutic strategies in refractory cases, as emphasized by the findings of Mollan et al. and Schmickl et al. in their studies [1-2].

Our study revealed that VSS is a safe procedure, with no significant complications. However, one patient experienced a temporary worsening of symptoms after the procedure, which subsequently resolved. This aligns with the findings of Townsend et al., who emphasized the importance of close monitoring in the immediate postoperative period [7].

Although our short- to medium-term outcomes are promising, VSS's long-term efficacy and safety remain unclear. Xu et al. stressed the need for extended follow-up periods to assess the persistence of treatment effects and potential long-term complications [6]. The longest follow-up was five months. Noting that conclusions regarding long-term efficacy or restenosis cannot be drawn from such short follow-up periods is important.

Our findings underscore the importance of appropriate patient selection based on clinical and radiological criteria because all patients in this series showed evidence of transverse sinus stenosis in imaging studies. This aligns with the recommendations of Ahmed et al. and Fargen et al. [4-14].

Our results are consistent with those of earlier studies that demonstrated the efficacy of VSS in reducing symptoms and improving patient quality of life [3,5,13]. The lower recurrence rate for VSS compared to medical therapy in our series aligns with the findings of Yeo et al. and Garner et al. [9-10].

As a small case series, our study has inherent limitations, including a small sample size, lack of a control group, varying follow-up periods, and absence of standardized outcome measures. These limitations are similar to those identified by Nicholson et al. in their systematic review and meta-analysis of VSS for IIH [15]. The varying follow-up periods significantly limited our ability to assess the long-term efficacy and complications. Future studies should standardize the follow-up period for at least one to provide more robust data on the long-term outcomes.

Recent research suggests that VSS may offer comparable or potentially superior outcomes to traditional surgical interventions, such as ventriculoperitoneal shunting and optic nerve fenestration, in select patients with IIH. VSS could provide effective symptom relief and pressure reduction with a lower complication rate, particularly in cases where venous sinus stenosis significantly contributes to IIH [16-17].

The positive outcomes observed in these patients likely resulted from the synergistic effect of VSS. They continued medical therapy after the procedure, highlighting the need for further research to isolate the specific effects of VSS. Future studies should standardize or withdraw medical therapy after VSS intervention, implement longer follow-up periods, and include control groups to provide more robust evidence of VSS efficacy as a stand-alone or complementary treatment.

Although our findings generally align with existing literature, they also raise new questions. For instance, the optimal timing of VSS in the course of IIH treatment and its potential role in preventing visual loss in early-stage disease remain unclear. Additionally, our results suggest a possible correlation between the degree of venous sinus stenosis and the treatment response, which warrants further investigation.

This case series provides preliminary evidence supporting VSS's potential benefits in the management of refractory IIH. While our findings are promising, they underscore the need for more extensive prospective studies with long-term follow-up periods to establish the role of the VSS in IIH management, as recommended by Fargen et al [13]. As our understanding of this procedure evolves, VSS may become an increasingly valuable tool for treating refractory IIH, potentially offering a paradigm shift in managing this challenging condition.

## Conclusions

This case series provides preliminary evidence regarding VSS as a management option for carefully selected patients with refractory IIH and documented venous sinus stenosis. While our observations align with the existing literature suggesting improvements in intracranial pressure, papilledema, visual symptoms, and headache following VSS, the conclusions that can be drawn are limited by the study design and scope. The small sample size, which included only four patients, the short follow-up duration of three to six months, and the concurrent use of acetazolamide in some patients precluded definitive statements regarding efficacy or long-term outcomes.

This case series highlights the need for tempering expectations regarding VSS durability based on this early clinical experience. Furthermore, while the procedure appeared safe in our limited experience, the lack of a control group limited our ability to draw firm conclusions regarding the safety profile of VSS relative to other treatment modalities. Future prospective controlled studies with longer follow-up periods are essential to definitively establish the comparative efficacy and long-term safety of VSS versus medical therapy or other surgical interventions for IIH. These studies should prioritize the optimization of patient selection criteria and investigate potential predictors of treatment response.
